# Against Milk Grading

**Published:** 1920-05-29

**Authors:** 


### THE PUBLIC WELL-BEING.

### AGAINST MILK GRADING.

### Suggestive Paper at Brussels Congress.

The Public-health Congress at Brussels seems to be proving a highly important gathering, and it is to be hoped that the English press will later on have access to a fuller record of the papers and discussions than is possible to expect from the cabled accounts. It is a pity that the Royal Institute of Public Health was unable to circulate papers in advance; it was the surest way of getting publicity for what must be of great value. The only really adequate report of any addresses that we have seen is that on municipal milk supply delivered by Mr. Shelmerdine (chairman of the Maternity and Infant-Welfare Committee of the Liverpool Corporation). This has been published in the *Manchester Guardian*, and was doubtless obtained by the paper's own enterprise because of Manchester's present discussion of the subject of municipal milk.

Mr. Shelmerdine, who initiated a system of sterilising milk by electricity, with which we hope to deal in a subsequent issue, made some interesting comments and suggestions, particularly in relation to the grading of milk, about which he expressed considerable disfavour. "I have little sympathy," he said, "with the American principle of grading milk into three classes, and then selling the higher grade as certified milk at an almost prohibitive price because it has a smaller bacterial content. All milk for sale to the public should be free from pathogenic bacteria, whether it is sold as market milk, cooking milk, or certified milk. As a matter of fact, most of the poorer and more ignorant classes look upon all milk simply as milk, disregarding dangers therefrom which they cannot see, and in actual practice the needy housewife buys the cheapest grade, rather than pay the higher price, and runs the risk, which she neither understands nor appreciates. Only one grade of water is admissible to the home; why should milk, which is much more to be feared, be admissible in three grades?" The only way to ensure a satisfactory milk supply (said Mr. Shelmerdine) is to begin at the cow, and it would be an advantage if it lay within the power of every urban authority to say to a producer wherever located:

"The milk sold within our jurisdiction must come from places that conform to certain requirements as determined by inspection by our own agents. If you desire to sell milk of this class, and none other can be sold in our city, we will, if you desire and request it, inspect your establishment for you." Municipal authorities generally have no power of compulsory inspection of those shippons at a distance from which their milk is derived, in order to ensure the healthfulness and cleanliness of cows, shippons, or farm hands, unless it is discovered by the merest chance that tuberculous milk is being sent into the city, and even then the greatest difficulty is experienced in locating and testing the individual cow, and this power of inspection is only possessed by very few English municipalities. What is very urgently wanted in the United Kingdom is a milk law, making it the duty of county councils, under a responsible Government Department, to make and enforce regulations to secure proper water supplies, drainage, ventilation, air space and cleaning of all shippons, dairies, and dairy farms within their districts; to secure the isolation of cattle suffering from contagious disease, and to carry into effect the provisions of the Act.

Preventive measures are better than corrective ones, but Mr. Shelmerdine is doubtful of the feasibility of the proper supervision of cowmen. How is it possible (he asks) to guarantee the cleanliness and cleanly action of the cowman who, in the early hours of the morning, proceeds, half-asleep, to milk the cows. When he enters the shippon the cows are recumbent, probably lying in their manure. Does he wait to groom the animals, to clear the manure from the flanks of the cows, or even wash his own hands before milking? There are undoubtedly clean cowmen, but generally the circumstances of the early hour and the distances between farms make official inspection and supervision impossible. Apart from the cleanliness of the cowman, the prevailing conditions regarding water supply at the farms in the United Kingdom for can washing and scalding and other purposes are usually of the poorest and most meagre description.

It had been urged against pasteurisation that it promotes carelessness and discourages efforts to produce clean milk, and tends to set back efforts in improvements at the source of supply; but in the meantime the public should undoubtedly be protected by the milk being made harmless.

Mr. Shelmerdine concluded by describing the beneficial results of the electrical method of treating milk as carried out in Liverpool.

